# Inheritance of Yield Components and Morphological Traits in Avocado cv. Hass From “Criollo” “Elite Trees” *via* Half-Sib Seedling Rootstocks

**DOI:** 10.3389/fpls.2022.843099

**Published:** 2022-05-24

**Authors:** Gloria Patricia Cañas-Gutiérrez, Stella Sepulveda-Ortega, Felipe López-Hernández, Alejandro A. Navas-Arboleda, Andrés J. Cortés

**Affiliations:** ^1^Corporación Colombiana de Investigación Agropecuaria AGROSAVIA, C.I. La Selva, Rionegro, Colombia; ^2^Corporation for Biological Research (CIB), Unit of Phytosanity and Biological Control, Medellín, Colombia

**Keywords:** *Persea americana* Mill., heritability, rootstock effects, seed-mediated grafting, “criollo” avocado tree

## Abstract

Grafting induces precocity and maintains clonal integrity in fruit tree crops. However, the complex rootstock × scion interaction often precludes understanding how the tree phenotype is shaped, limiting the potential to select optimum rootstocks. Therefore, it is necessary to assess (1) how seedling progenies inherit trait variation from elite ‘plus trees’, and (2) whether such family superiority may be transferred after grafting to the clonal scion. To bridge this gap, we quantified additive genetic parameters (i.e., narrow sense heritability—*h*^2^, and genetic-estimated breeding values—GEBVs) across landraces, “criollo”, “plus trees” of the super-food fruit tree crop avocado (*Persea americana* Mill.), and their open-pollinated (OP) half-sib seedling families. Specifically, we used a genomic best linear unbiased prediction (*G*-BLUP) model to merge phenotypic characterization of 17 morpho-agronomic traits with genetic screening of 13 highly polymorphic SSR markers in a diverse panel of 104 avocado “criollo” “plus trees.” Estimated additive genetic parameters were validated at a 5-year-old common garden trial (i.e., provenance test), in which 22 OP half-sib seedlings from 82 elite “plus trees” served as rootstocks for the cv. Hass clone. Heritability (*h*^2^) scores in the “criollo” “plus trees” ranged from 0.28 to 0.51. The highest *h*^2^ values were observed for ribbed petiole and adaxial veins with 0.47 (CI 95%0.2–0.8) and 0.51 (CI 0.2–0.8), respectively. The *h*^2^ scores for the agronomic traits ranged from 0.34 (CI 0.2–0.6) to 0.39 (CI 0.2–0.6) for seed weight, fruit weight, and total volume, respectively. When inspecting yield variation across 5-year-old grafted avocado cv. Hass trees with elite OP half-sib seedling rootstocks, the traits total number of fruits and fruits’ weight, respectively, exhibited *h*^2^ scores of 0.36 (± 0.23) and 0.11 (± 0.09). Our results indicate that elite “criollo” “plus trees” may serve as promissory donors of seedling rootstocks for avocado cv. Hass orchards due to the inheritance of their outstanding trait values. This reinforces the feasibility to leverage natural variation from “plus trees” *via* OP half-sib seedling rootstock families. By jointly estimating half-sib family effects and rootstock-mediated heritability, this study promises boosting seedling rootstock breeding programs, while better discerning the consequences of grafting in fruit tree crops.

## Introduction

Grafting is an ancient technique used for vegetative propagation, especially in perennial fruit crops. This method, used on woody and herbaceous plants, can improve several agronomic characteristics, such as yield and vigor, as well as tolerance to biotic and abiotic stresses (Loupit and Cookson, 2020). In fruit trees, grafting is a common propagation method because it provides a dual plant system to increase orchard productivity by maintaining genetic uniformity of the commercial clones. Usually, scions’ buds with high quality are grafted onto rootstocks with improved stress/disease tolerance. In this dual plant system, rootstocks are also selected to increase orchard efficiency through vigor control and yield improvement of the scion. Increasing precocity is another benefit of grafting, as scions taken from mature trees show significantly earlier bearing and maturity relative to trees grown from seeds. Therefore, many tree crops with long juvenile phases are grown as grafted plants to obtain a faster return on investment (Ahsan et al., 2019). Despite these benefits, grafting tends to obscure individual genotypic contributions from the scion and the rootstock to the overall tree phenotype, which makes genetic selection harder at particular rootstock × scion combinations.

From a physiological perspective, such confounding factors are pervasive, too. Broadly speaking, grafting affects three main processes at the tree level: uptake and transport of water and nutrients (Little et al., 2016), production and transport of hormones, and large-scale movement of proteins, messenger RNAs, and small RNAs (Wang et al., 2017; Loupit and Cookson, 2020; Lu et al., 2020; Rasool et al., 2020). These processes have implications for both subsurface and surface functioning, yet the interconnection of variables at the rootstock—scion interface still hides contributions from the individual genotypes (Tworkoski and Miller, 2007; Amiri et al., 2014; Warschefsky et al., 2016). After all, both rootstock and scion genotypes play an important role at the grafting interface, and different rootstock × scion combinations mutually alter their individual phenotypic effects (Goldschmidt, 2014). Additional factors that may affect the rootstock × scion interactions are the age of the bud-donor tree, the grafting technique, seasonality, time since grafting, the genotype × environment interaction (Albacete et al., 2015), the rootstock × scion compatibility, and microbiome-root interactions (Warschefsky et al., 2016).

A highly valued grafted clonal super-food fruit tree crop that is rapidly expanding around the world is avocado (*Persea americana* Mill.) cv. Hass (O’Brien et al., 2018). Improved rootstocks for commercial avocado cv. Hass plantations may confer a beneficial horticultural quality to the tree across a wide spectrum of traits (Reyes-Herrera et al., 2020), such as increased fruit yield (Herrera-González et al., 2013), postharvest performance (Willingham et al., 2001), vegetative vigor (Mickelbart and Arpaia, 2002), salt tolerance (Bernstein et al., 2001), and disease resistance (Smith et al., 2011; Sánchez-González et al., 2019). These reports are in line with previous research that have shown how rootstocks might also induce less trivial scion morphological changes, such as dwarfing, and even alter yield traits and fruit quality (Egea et al., 2004; Picolotto et al., 2010; Madam et al., 2011; Expósito et al., 2020; Kviklys and Samuoliene, 2020). For instance, rootstock effects may even influence properties typically attributed to the clonal Hass scion, such as fruit sensorial and nutritional quality, e.g., texture, sugar content, acidity, pH, flavor, and color (Giorgi et al., 2005; Gullo et al., 2014; Balducci et al., 2019), cold tolerance, and shoot pest and pathogen resistance (Rubio et al., 2005; Goldschmidt, 2014).

Most commercial avocado Hass plantations in Neotropical areas currently rely on open-pollinated (OP) half-sib interracial seedling rootstocks derived from selected “criollo” “plus trees” (Bernal et al., 2014; Cañas-Gutiérrez et al., 2015). Therefore, avocado production is mainly based on grafting the commercial Hass cultivar onto untested highly diverse seedling “criollo” saplings (Rodríguez et al., 2009; Cañas-Gutiérrez et al., 2015). As part of this procedure, the selection of a suitable rootstock is rarely based on both the genotype of the scion, and the environment or agro-climatic zone in which the grafted tree will be cultivated. In other words, due to a triple rootstock × scion × environment interaction, rootstock selection from “criollo” seedling genotypes is challenging. Still, growing half-sib families of seedling rootstocks from selected donor “plus trees” remains a promising strategy because they may harbor natural adaptations to the highly heterogeneous ecosystems found at the Neotropics, not to mention the fact that they constitute an important source of genetic variability (Kuhn et al., 2019; Rendón-Anaya et al., 2019; Talavera et al., 2019; López-Guzmán et al., 2021) for otherwise clonal Hass plantations (Cañas-Gutiérrez et al., 2019a).

Avocado rootstock breeding programs typically perform an initial selection step for resistance to soil-borne pathogens, such as *Phytophthora cinnamomi* Rans. Yield and adaptive traits are then left as second and third steps within avocado rootstock breeding. To guarantee that selected rootstocks exhibit a stable phenotypic effect, avocado nurseries in temperate latitudes often rely on clonal rootstock propagation (Téliz, 2000). However, clonal rootstocks are still difficult to produce because they require double grafting techniques (Ernst, 1999), while *in vitro* production has not been commercially scaled (Hormaza, 2020). Therefore, clonal rootstocks are still rare in highly diverse Neotropical regions where avocados are native, and seedling saplings from “criollo” trees are more abundant and cheaper. Because of this, it is imperative to assess which traits of the avocado “criollo” are inherited to its OP half-sib seedling progenies, while at the same time transferred to the scion after grafting. Specifically, an explicit estimation of combined half-sib families and rootstock heritability effects would be a major advance to speed-up seedling rootstock breeding programs, and ultimately discern the consequences of grafting for tropical avocados (Reyes-Herrera et al., 2020).

To fill this research gap, the goals of this work were to: (1) estimate additive genetic parameters (i.e., narrow sense heritability scores and breeding values) across promissory avocado “criollo” “plus trees,” (2) quantify the inheritance of agronomic traits of avocado “criollo” “plus trees” in cv. Hass *via* OP half-sib seeding families, and (3) identify promissory avocado “criollo” “plus trees” as seed donors for rootstock production based on their genetic-estimated breeding values for heritable morphological and agronomic traits. We hypothesize that heritable variation for key traits would enable half-sib seedling rootstock selection from elite “criollo” “plus trees.” Specifically, we have relied on a total of 104 avocado “criollo” trees between 40 and 50 years old, identified as “plus trees” in the province of Antioquia (northwest Colombia). These avocado trees are long-term survivors across different remote agro-ecological zones, so we expect that they may serve as potential donors of seedling rootstocks due to their presumably outstanding adaptability and trait values (e.g., health, longevity, and productivity). These “criollo” avocados had previously been characterized with microsatellite (single sequence repeat, SSR) markers to study their genetic diversity, as well as in terms of their morpho-agronomic traits (Cañas-Gutiérrez et al., 2019a,b). Therefore, this study merged both genetic and phenotypic characterizations to assist seedling rootstock selection at early nursery stages. Estimated additive genetic parameters were validated at a 5-year-old common garden trial (i.e., provenance test) that established OP half-sib seedlings from elite “plus trees” as rootstocks for the Hass clone.

## Materials and Methods

### Plant Material

A total of 104 avocado “criollo” “plus trees” were selected in different remote agroecological zones up to 2,400 m asl of the province of Antioquia, northwest Andes of Colombia, during the years 2014 and 2015 (originally enlisted under the first table of Cañas-Gutiérrez et al. (2019b), and summarized here under Supplementary Table 1). Their selection was made based on criteria, such as adaptability to their growing area, longevity, health, and productivity. During this period, trees were *in situ* characterized by taking morphological data, and carrying out their molecular fingerprinting (section below on “microsatellite markers characterization”). Collection of yield data was additionally carried out in these trees during four consecutive years (2014–2017).

The seeds produced by selected elite avocado “criollo” trees were germinated at greenhouses, and obtained 3-month-old seedlings were grafted with cv. Hass scions. In the year 2015, the cv. Hass grafts were planted at a common garden (i.e., provenance test) at AGROSAVIA’s research station La Selva, located in Rionegro, province of Antioquia, at 2,100 m asl. Morphological data, such as length and diameter of the rootstock, the canopy length and diameter of the scion, and the morphology of the graft scar were collected during the years 2017–2021. The production data of the cv. Hass grafts were collected in the same period from 2017 to 2021. Since half-sib seedling rootstocks grafted with the Hass clone have been in production during five consecutive years in the same environment (La Selva locality), we were able to minimize environmental variance and equal phenotypic variance to the rootstocks’ additive genetic component, which is the main benefit of carrying out common garden or provenance tests. These trials intend to neutralize environmental effects over phenotypic expression, by planting diverse genotypes in a single locality (Lascoux et al., 2016). Our setup has allowed heritability analyses to include a total of 9 years of production data, both for *in situ* “criollo” “plus trees” (2014–2017), as well as in their OP half-sib seedling progenies used as rootstocks for the Hass scion (2017–2021).

### Measurements of Morpho-Agronomic Traits

**FIGURE 1:**
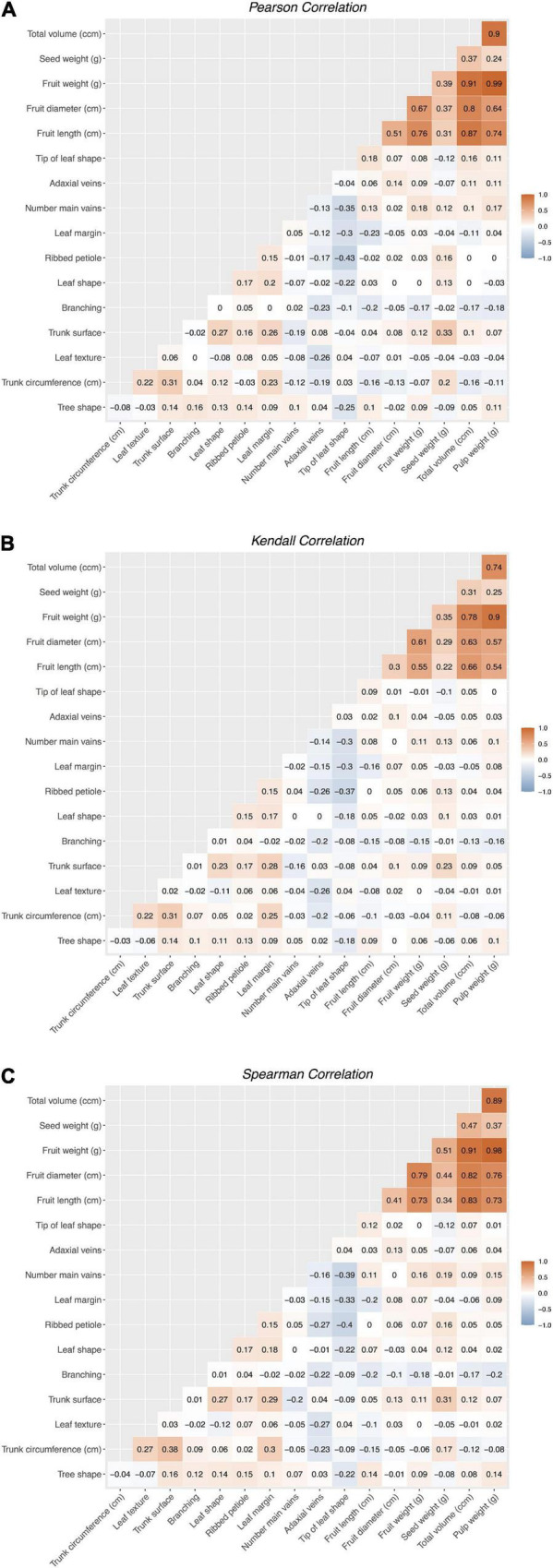
Pearson **(A)**, Spearman **(B)**, and Kendall **(C)** correlation matrices among studied traits in avocado “criollo” “plus trees.” For details on the measured traits please refer to Table 1 and Supplementary Table 2.

A total of 17 morphological and agronomic traits were measured during the sampling trips of 2014 and 2015 in the avocado “criollo” selected “plus trees,” according to descriptors that are specific for avocado based on the International Plant Genetic Resources Institute (IPGRI). These descriptors included tree, leaf, fruit, and seed traits (Table 1 and Supplementary Table 2). Fruit production was recorded in these trees during four consecutive years until 2017. Pearson’s, Spearman’s and Kendall’s correlation matrices (Figure 1) were visually inspected using customized plots drawn with the *cor.test* and *heatmap* functions in R v.3.4.4 (R Core Team). Complete phenotypic measures were possible for a total of 82 “criollo” “plus trees” from the original sampling.

**TABLE 1:** Phenotypic traits measured in the avocado “criollo” selected “plus trees,” according to descriptors for avocado of the International Plant Genetic Resources Institute (IPGRI).

Type of trait	Phenotypic traits	Abbreviation	Units of measurement
Morphological traits	Tree shape	TSH	Qualitative
	Trunk circumference	TC	Centimeters
	Trunk surface	TS	Qualitative
	Branching	B	Qualitative
	Leaf texture	LT	Qualitative
	Leaf shape	LS	Qualitative
	Leaf margin	LM	Qualitative
	Tip of leaf shape	TLS	Qualitative
	Number main veins	NMV	Number
	Adaxial Veins	AV	Qualitative
	Ribbed petiole	RP	Qualitative
Agronomic traits	Fruit length	FL	Centimeters
	Fruit diameter	FD	Centimeters
	Fruit weight	FW	Grams
	Seed weight	SW	Grams
	Pulp weight	PW	Grams
	Total volume	TV	Grams

*For details on quality measures and their codifications refer to Supplementary Table 2.*

Furthermore, a total of 22 OP half-sib seedling progenies of 82 elite avocado “criollo” “plus trees” were used as rootstocks for the Hass scion. Grafted trees were characterized at AGROSAVIA’s La Selva research station in terms of their early growth traits (i.e., young shoot color, stem color, stem lenticels, lenticels color, average number of leaves at transplant, and average stem diameter at grafting), as well as in terms of their production (i.e., cumulative total number of fruits and fruit’s weight) 5 years after grafting (2017–2021). The analyses focused on yield traits because they allowed for a direct comparison with the recorded production traits in the “criollo” “plus trees,” besides the fact that yield traits are of paramount interest for commercial avocado cv. Hass orchards, especially those aiming for exportation markets.

### Microsatellite Marker Characterization

Total genomic DNA was obtained from avocado “criollo” leaves based on the extraction method standardized by Cañas-Gutiérrez et al. (2015). Thirteen microsatellites [simple sequence repeats (SSRs)], originally designed by Sharon et al. (1997) and Ashworth et al. (2004), were chosen (Supplementary Table 3) for their high polymorphism information content following estimates by Alcaraz and Hormaza (2007). Fingerprinting highly polymorphic SSR markers allowed disclosing pedigree-free marker-based relationships among genotypes, which in turn enabled distinguishing from highly related to completely unrelated individuals. Such contrasting scales of SSR-reconstructed shared relatedness due to recent co-ancestry (i.e., isolation by descendent, IBD) offer the basis to quantify phenotypic clustering patterns across family types (i.e., genetic heritability), as demonstrated in oil palm (Cros et al., 2015) and rubber tree (Cros et al., 2019), and as expanded in the section below.

Three multiplex PCR amplifications were performed in 10 μl volume containing 16 mM (NH4)2SO4, 67 mM Tris-HCl pH 8.8, 0.01% Tween20, 2 mM MgCl2, 0.1 mM each dNTP,0.4 μM of each primer, 25 ng genomic DNA and GoTaq^®^ ADN polymerase (Promega, WI, United States). Forward primers were labeled with WellRed fluorescent dyes on the 5′ end (Proligo, France). The reactions were carried out in an I-cycler (Bio-Rad Laboratories, Hercules, CA, United States) thermo cycler using the following temperature profile for multiplex PCR 2 and 3: an initial step of 1 min at 94°C, 35 cycles of 30 s at 94°C, 30 s at 50°C, and 1 min at 72°C, and a final step of 5 min at 72°C. For multiplex PCR 1, the same temperature profiles were used, except for 55°C for annealing temperature that was used instead. The PCR products were analyzed by capillary electrophoresis in the equipment ABI PRISM^®^ 3130 Genetic Analyzer (Applied Biosystem, CA, United States). Allele sizes were estimated in base pairs with a Peak Scanner (Thermo Fisher Scientific, United States), allowing for a maximum of two alleles per sample.

### Computation of Heritability Scores and Breeding Values

We implemented a *G*-BLUP (genomic best linear unbiased predictor) model to quantify genetic parameters (i.e., narrow-sense heritability and breeding values) for key agronomic traits across the avocado “criollo” “plus trees” selected as seed donors for the OP half-sib progenies used as rootstocks for the Hass clone. Genetic parameters are a baseline of any breeding program (Holland et al., 2003) since they guarantee that additive genetic gains are maximized, while breeding cycles are minimized (Dieters et al., 1995). This is because genetic parameters account for the proportion of phenotypic variance among individuals in a population due to genetic effects (Milner et al., 2000; Kruuk, 2004; Berenos et al., 2014). In this sense, the *G*-BLUP precisely offers a modern pedigree-free marker-based approach (Meuwissen et al., 2001; Crossa et al., 2017) to estimate genetic parameters on populations of mixed ancestry (Frentiu et al., 2008; Wilson et al., 2010), such as OP seedlings.

We first computed a *G* relatedness matrix, following Lynch and Ritland (1999), to account for the half-sib structure of the OP seedling progenies used as rootstocks. To validate relationships within the *G* matrix we further computed (1) a distance tree by means of the Neighbor Joining algorithm (Kamvar et al., 2014) in R (R Core Team) with 10,000 bootstrap replicates, as well as (2) an unsupervised Bayesian clustering using the STRUCTURE software (Pritchard et al., 2000) with five independent runs for each *K*-value from *K* = 2 to *K* = 8 and 100,000 Monte Carlo Markov chain replicates with a burn-in of 50,000.

The admixed origin of the avocado “criollo” genepool, due to avocado’s protogynous dichogamy, is known to favor an adequately variable *G* matrix, embracing various degrees of relationship, as much as the Avocado Genebank (Cañas-Gutiérrez et al., 2019b). The variability in the *G* matrix (from highly related to completely unrelated trees) enabled the use of the *G*-BLUP predictor. Hence, we relied on this *G* relationship matrix to compute the genetic-based *G*-BLUP model, following Arenas et al. (2021) and Fernandez-Paz et al. (2021), as in:


y=X⁢b+Z⁢a+e

where *y* corresponds to the phenotypic trait vector, *a* is the vector of individual random additive genetic effects with a normal distribution [in other words, *a* ∼ N (0, *G σ_*a*_^2^*), where *G* is the among-tree pedigree-free SSR-based relationship matrix, and *σ_*a*_^2^* corresponds to the additive genetic variance (Müller et al., 2017)], *b* is the vector of fixed effects (i.e., general mean, intercept), *e* is the vector of residual effects, and *X* and *Z* are the incidence matrices for fixed effects and additive genetic effects (Chen et al., 2018; Gutierrez et al., 2018).

Parameter estimation for the *G*-BLUP model was carried out using reproducing kernel Hilbert space (RKHS) implemented in the BGLR (Bayesian Generalized Linear Regression) software (Perez and De Los Campos, 2014) under an R v.3.4.4 environment (R Core Team). The multi-dimensional space of parameters was sampled with 10,000 iterations, an initial burn-in of 5,000 steps, and a thinning interval of 10 for data recording. Trace plots were drawn in R to verify convergence in the posterior distributions.

Narrow sense rootstock-mediated heritability (*h*^2^), which is the proportion of the overall phenotypic variance accounted for additive genetic variance, was then calculated by retrieving the additive (*σ_*a*_*^2^) and residual (*σ_*e*_*^2^) variances from the RKHS algorithm, following de los Campos et al. (2015):


$$h^{2}=\sigma_{a}^{2}/\left(\sigma_{a}^{2}+\sigma_{e}^{2}\right)$$

The vector of *h*^2^ scores was summarized using the median and the 95% CI of the BGLR’s posterior distribution. Statistical accuracy (a.k.a., prediction ability, *r*_*y*_) was computed per trait using Pearson’s correlation between observed and predicted (μ + GEBV) trait values (Müller et al., 2017; Zhang et al., 2019). Predicted trait values were recorded and subtracted to the overall phenotypic mean (μ), to gather the vector of genetic-estimated breeding values (GEBVs), which is the deviation from the overall trait mean attributed to additive genetic effects. GEBVs were summarized throughout a biplot diagram using R’s *princomp* and *biplot* functions for the principal components analysis (PCA).

Finally, we implemented mother-offspring regressions to estimate *h*^2^ scores for key agronomic traits in the OP half-sib seedling progenies of avocado “criollo” “plus trees” grafted with the Hass scion. This heritability analysis included 5 years of data in the OP half-sib seedling progenies of avocado “criollo” “plus trees” used as rootstocks for the Hass scion *via* the mother vs. half-sib regression analysis (Falconer et al., 1996). These *h*^2^ estimates were gathered for cumulative number of fruits and weight of fruits from the Hass clone grafted on top of the OP half-sib seedling rootstocks derived from the selected avocado “criollo” “plus trees.” Mother-offspring regressions were carried out in R using the function *lm*. Regressions were plotted for validation in *ggplot*, under the same R environment. The narrow-sense heritability score was calculated as twice the slope from the mother-offspring regressions, following Lynch and Walsh (1998).

## Results

### Heritability of Morphological and Agronomic Traits in Avocado “Criollo” “Plus Trees”

**FIGURE 2:**
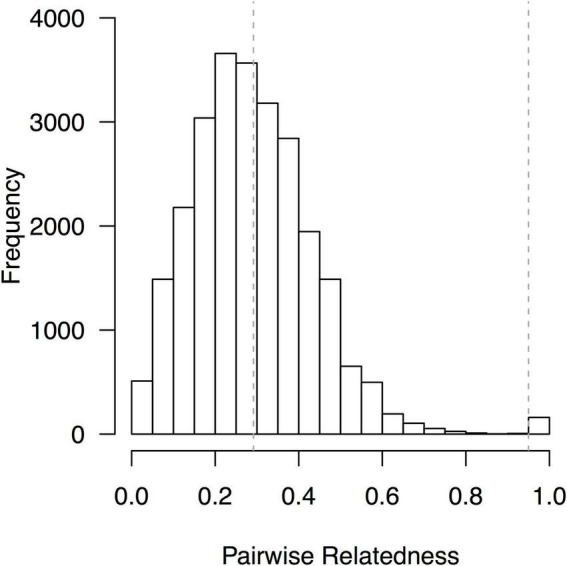
Pairwise relatedness among avocado “criollo” “plus trees” according to Lynch and Ritland (1999). The overall mean and the 0.95 threshold to identify genetic controls are marked with dashed vertical lines.

**FIGURE 3:**
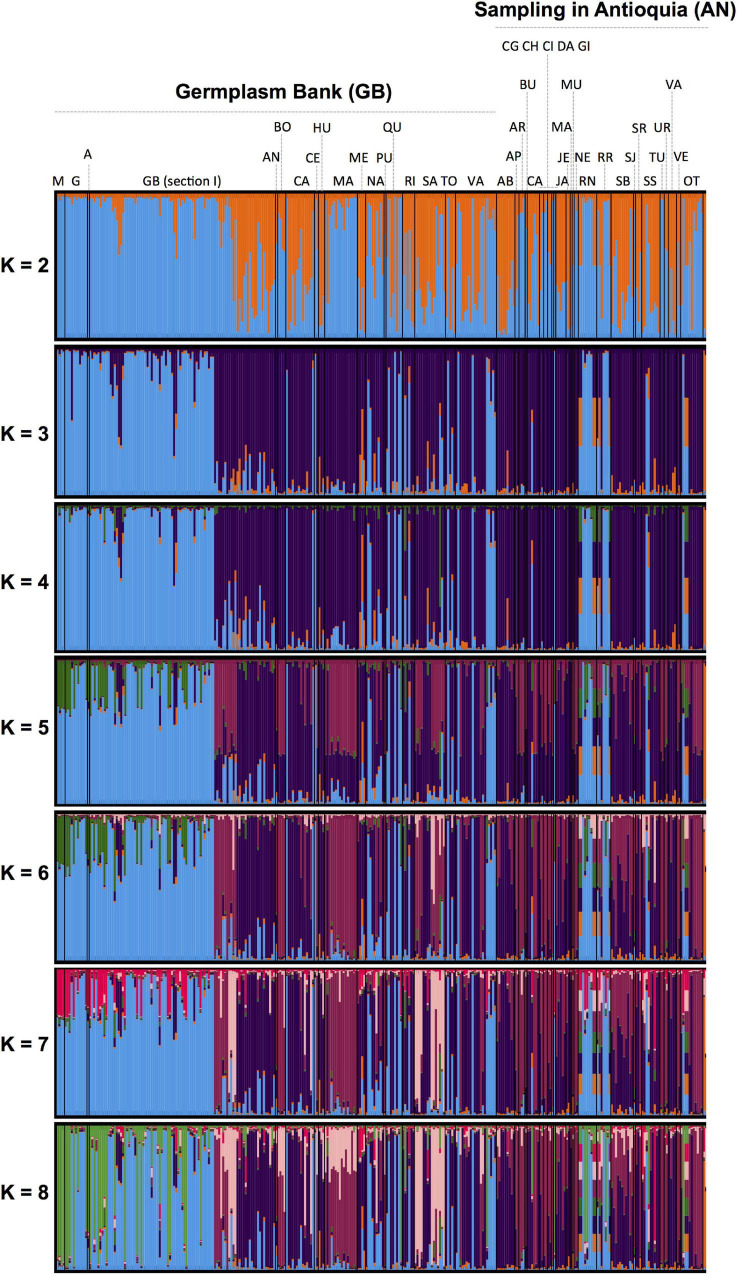
Unsupervised Bayesian clustering among avocado “criollo” “plus trees” collected from the northwest Andes of Colombia (province of Antioquia, AN), and the avocado Germplasm Bank (GB) from Colombia as characterized by Cañas-Gutiérrez et al. (2019a) with the same panel of SSR markers. M, G, and A, stand for Mexican, Guatemalan, and West Indies race controls, respectively. GB Section 1 comprises commercial genotypes. The remaining two-letter abbreviation codes indicate avocado “criollo” trees, respectively sampled from different provinces (under GB) and villages (under AN) across Colombia and the province of Antioquia (northwest Andes of Colombia), as follows: Antioquia (AN), Bolivar (BO), Cauca (CA), Cesar (CE), Huila (HU), Magdalena (MA), Meta (ME), Nariño (NA), Putumayo (PU), Quindío (QU), Risaralda (RI), Santander (SA), Tolima (TO), and Valle del Cauca (VA) for provinces across Colombia, and Abejorral (AB), Apartadó (AP), Arboletes (AR), Buriticá (BU), Caramanta, Carepa or Caracolí (CA), Cañas Gordas (CG), Chigorodó (CH), Cisneros (CI), Dabeiba (DA), Giraldo (GI), Jardín (JA), Jericó (JE), Maceo (MA), Mutatá (MU), Necoclí (NE), Rionegro (RN), Urrao (RR), Santa Bárbara (SB), San Juan (SJ), San Roque (SR), Sonsón (SS), Turbo (TU), Uramita (UR), Valparaiso (VA), Venecia (VE), and other (OT) for municipalities within the province of Antioquia. For further details on the sampling within Antioquia, such as sub-region and elevation, please refer to Supplementary Table 1 and the first table in Cañas-Gutiérrez et al. (2019b).

The 13 microsatellite markers that were used for the molecular characterization of 104 avocado “criollo” “plus trees” amplified a total of 147 allelic fragments. This indicated sufficient polymorphism to reconstruct the pedigree-free marker-based relationship matrix (Figure 2), while distinguishing highly related from completely unrelated admixed individuals (Figure 3). The phylogenetic tree (Supplementary Figure 1) corroborated the latter point. Therefore, the segregation of 147 allelic SSR fragments allowed to effectively quantify phenotypic clustering patterns across family types *via* de *G*-BLUP model (i.e., SSR-based genetic heritability estimates, Cros et al., 2015, 2019) for key morphological and agronomic traits in “criollo” avocado “plus trees.”

**FIGURE 4:**
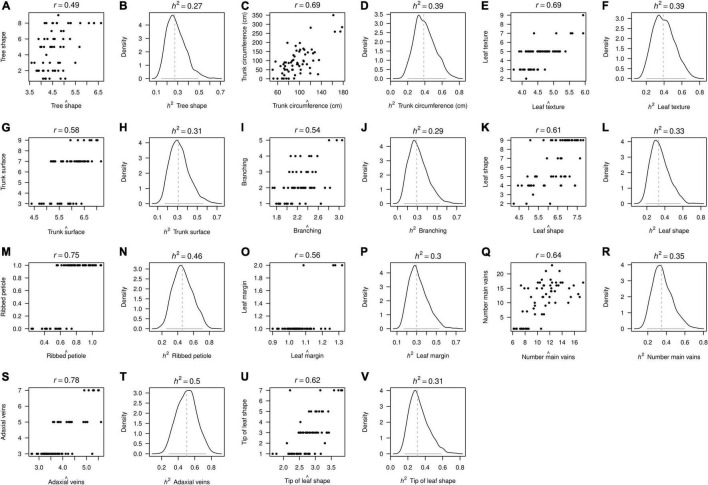
Narrow-sense heritability (*h*^2^*)* posterior distributions for morphological traits measured in avocado “criollo” “plus trees” selected as seed donors for rootstocks of the cv. Hass. Model fits are depicted in the odd columns **(A,C,E,G,I,K,M,O,Q,S,U)** using Pearson’s correlation *r* score between the observed (y-axis) and the predicted (x-axis) trait values. Meanwhile, median values (vertical dashed lines) and the 95% confidence intervals (horizontal gray lines) are depicted in the even columns **(B,D,F,H,J,L,N,P,R,T,V)**. Different traits are sorted every second subpanel across rows according to the x-label in each subfigure.

**FIGURE 5:**
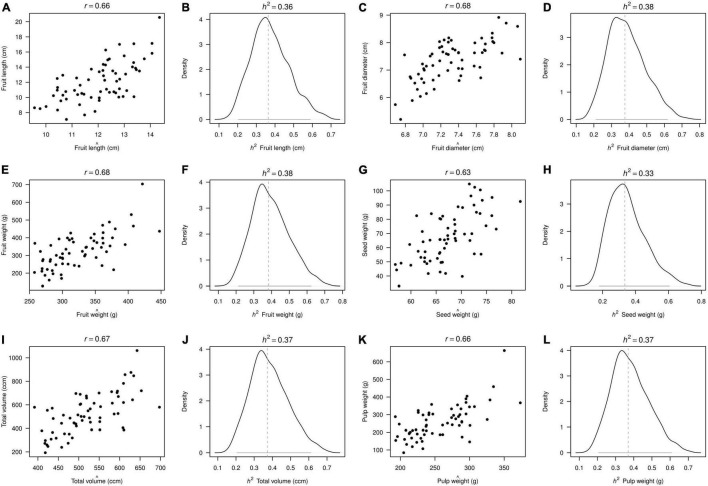
Narrow-sense heritability (*h*^2^) posterior distributions for agronomic traits measured in avocado “criollo” “plus trees” selected as seed donors for rootstocks of the cv. Hass. Model fits are depicted in the odd columns **(A,C,E,G,I,K)** using Pearson’s correlation *r* score between the observed (y-axis) and the predicted (x-axis) trait values. Meanwhile, median values (vertical dashed lines) and the 95% confidence intervals (horizontal gray lines) are depicted in the even columns **(B,D,F,H,J,L)**. Different traits are sorted every second subpanel across rows according to the x-label in each subfigure.

**FIGURE 6:**
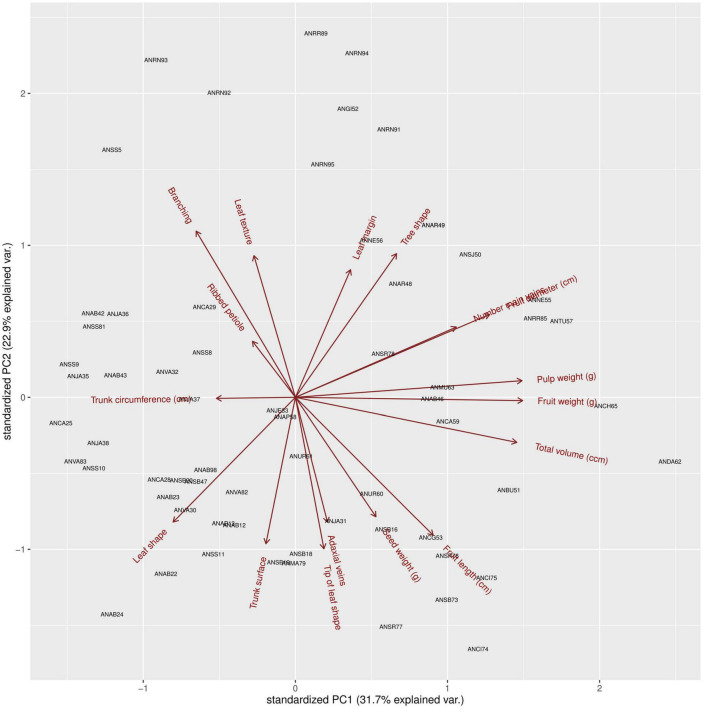
Breeding values for traits (vectors in red) measured in avocado “criollo” “plus trees” (black codes) selected as seed donors for rootstocks of the cv. Hass. A match between the specific tree code and the arrow of the trait vector indicates an additive genetic superiority (a.k.a., breeding value) of that particular tree for the specific trait. Opposing vectors speak for negative genetic correlations, which may limit simultaneous selection for both traits in the same direction, as opposed to orthogonal vectors that suggest traits are free from genetic correlations.

The heritability parameters for a total of 11 morphological traits (Figure 4) and 6 agronomical traits (Figure 5) were computed in 82 of the 104 avocado “criollo” “plus trees” selected as seed donors for rootstocks of the cv. Hass. Estimates of avocado “criollo” “plus trees” narrow-sense heritabilities (*h*^2^) were significant for the 11 measured morphological traits and for the 6 agronomic traits, according to the 95% CIs from the posterior distribution, all of which were above absolute zero (Table 2).

**TABLE 2:** Narrow—Sense heritability (*h*^2^*)* estimates for the 17 measured traits from 82 avocado “criollo” “plus trees” selected as seed donors for rootstocks.

	Phenotypic traits	*h* ^2^	IC_95%_	*r*
Morphological traits	Tree shape	0.27	0.15–0.46	0.49
	Trunk circumference (cm)	0.39	0.20–0.64	0.69
	Trunk surface	0.31	0.16–0.53	0.58
	Branching	0.29	0.16–0.54	0.54
	Leaf texture	0.39	0.20–0.60	0.69
	Leaf shape	0.33	0.18–0.55	0.61
	Leaf margin	0.30	0.16–0.51	0.56
	Tip of leaf shape	0.31	0.15–0.58	0.62
	Number main veins	0.35	0.19–0.58	0.64
	Adaxial veins	0.50	0.26–0.72	0.78
	Ribbed petiole	0.46	0.26–0.71	0.75
Agronomic traits	Fruit length (cm)	0.36	0.19–0.58	0.66
	Fruit diameter (cm)	0.38	0.21–0.60	0.68
	Fruit weigh (g)	0.38	0.22–0.60	0.68
	Seed weight (g)	0.33	0.18–0.61	0.63
	Pulp weight (g)	0.37	0.21–0.58	0.66
	Total volume (cm^2^)	0.37	0.20–0.59	0.67

*Narrow-sense heritability (h^2^) and model fits (Pearson’s r between the observed and the predicted trait value) estimates were gathered using Lynch and Ritland’s (1999) relatedness matrix inputted in a “genetic prediction” additive mixed linear model, according to de los Campos et al. (2009). The significance of heritability scores was determined according to the 95% confidence intervals from posterior distributions, all of which were above the absolute zero.*

The estimated heritabilities (*h*^2^) for the 11 morphological traits ranged from 0.28 to 0.51. The tree shape and branching traits presented the *h*^2^ lowest scores, 0.28 and 0.29, respectively. The highest *h*^2^ values were presented for ribbed petiole and adaxial veins with 0.47 and 0.51, respectively. The 6 agronomic traits evaluated presented very similar *h*^2^ values that ranged from 0.34 to 0.39. Seed weight was the trait with the lowest *h*^2^ score (0.34), while fruit weight and total volume traits exhibited the highest values, both with 0.39 (Table 2).

Confidence intervals for the heritability scores were generally narrow for the 17 examined traits. The traits that presented the widest confidence intervals were: number main veins (0.1–0.7, *r* = 0.64) pulp weight (0.1–0.7, *r* = 0.66), adaxial veins (0.2–0.8, *r* = 0.78), and ribbed petiole (0.2–0.8, *r* = 0.75). Meanwhile, the trait that presented the narrowest confidence interval was the shape of the tree with 0.1–0.4 and *r* = 0.69 (Table 2). Adaxial veins and ripped petiole traits exhibited the widest 95% CIs, as well as the highest heritability scores (0.51 and 0.47, respectively). On the contrary, the tree shape trait had the lowest 95% CI, and also showed the lowest heritability score (0.28). Broadly speaking, the morphological and agronomic traits measured in avocado “criollo” “plus trees” selected as seed donors for rootstocks of the cv. Hass presented heritabilities in a medium range. This suggested a relative contribution of additive genetic effects in the total variation of these traits, which may enable moderate genetic gains *via* recurrent selection.

### Breeding Values of Avocado “Criollo” “Plus Trees” to Select Target Seed Donors

To assist the ranking of avocado “criollo” “plus trees” as seed donors for OP half-sib seedling rootstocks of the cv. Hass, we summarized the breeding values for the 11 morphological and 6 agronomical traits across 63 of the 104 avocado “criollo” “plus trees” selected as seed donors for rootstocks of the cv. Hass using a biplot diagram from a PCA (Figure 6). The length of each vector in the biplot diagram allowed ranking the traits by their additive variance. In this sense, fruit diameter, total volume, fruit weight, pulp weight, fruit length, and branching exhibited the most notorious additive genetic contributions, reinforcing the heritability results described in the previous section. On the contrary, the ribbed petiole and trunk circumference traits, showed lower vector lengths, indicating a lower additive genetic variance for these traits. These results suggested that fruit traits tended to exhibit more potential to respond to selection, indicating that production at commercial avocado cv. Hass orchards may be leveraged by a proper selection of avocado “criollo” “plus trees” as seedling rootstock donors, based on their breeding values.

The biplot diagram also allowed pinpointing the elite avocado “criollo” trees that may be promising for selection given their heritable superiority in terms of agronomical traits. For instance, the trait vector for fruit diameter (associated with the fruits’ caliber, an important factor for its commercialization) pointed toward “criollo” trees ANSS9 and ANNE55. Similarly, the trait vectors for total fruits’ volume, fruits’ length, and fruits’ weight, respectively pointed toward the avocado “criollo” “plus trees” encoded as ANDA62, ANCI75, and ANCH65. Meanwhile, the avocado “criollo” tree encoded as ANTU57 was closed to the vectors for the traits fruit diameter and pulp weight, which also makes ANTU57 a promising donor for seedling rootstock families (Figure 6). On the other hand, the morphological traits branching, leaf shape, trunk surface, tree shape, and adaxial veins presented the longest vectors, and consequently greater potential for selection. Some of these morphological traits may be of interest for half-sib rootstock family selection. For example, branching and tree shape may contribute desired tree architectures optimum for light penetration to boost flowering and harvesting.

### Inheritance of Key Agronomic Traits on the Hass Scion Through Open-Pollinated Half-Sib Seedling Rootstocks of Avocado “Criollo” “Plus Trees”

**FIGURE 7:**
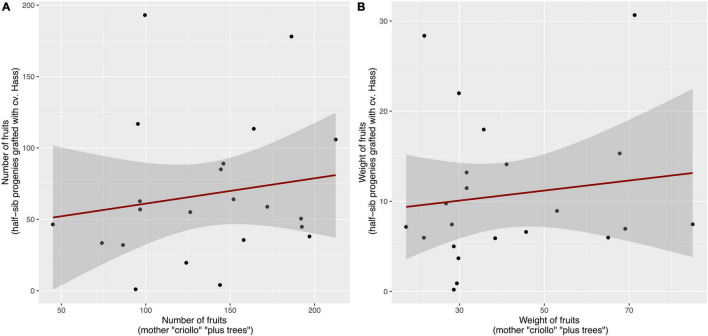
Mother-offspring regressions used to quantify the rootstock-mediated *h*^2^ scores for cumulative **(A)** number of fruits and **(B)** weight of fruits from the Hass clone grafted on OP half-sib seedling progenies of selected avocado “criollo” “plus trees.” The *h*^2^ score is computed as twice the slope from the mother-offspring regression (Lynch and Walsh, 1998), and was 0.36 and 0.11 *h*^2^ for number of fruits and weight of fruits, respectively.

A total of 22 “criollo” “plus trees” were selected as seed donors for rootstock establishment according to the trait criteria enlisted in the previous section. Their corresponding OP half-sib seedling progenies were grafted with Hass clonal scion to estimate rootstock-transferred *h*^2^ scores for key agronomic traits, such as total number of fruits and fruits’ weight after 5 years of production. The estimation of overall *h*^2^ scores (spanning both the mother-offspring and the rootstock-mediated inheritance) for both agronomical traits followed a mothers vs. grafted half-sib offspring linear regression. In this sense, the dependent variable corresponded to the average half-sib family trait values for total number of fruits and fruits’ weight measured in avocado cv. Hass trees grafted on top of OP seedling rootstock families obtained from the avocado “criollo” “plus trees.” The trait values for the total number of fruits and fruits’ weight measured in the original mother “criollo” “plus trees” were depicted in the *X*-axis (Figure 7).

The trait total number of fruits presented a slope of 0.18 and an *r* score of 0.16. Since the mothers-offspring heritability corresponded to twice the slope, the *h*^2^ equaled 0.36 (± 0.23). The trait fruits’ weight exhibited a slope value of 0.056 with an *r* score of 0.18, and therefore an overall *h*^2^ estimate of 0.11 (± 0.09, Figure 7). According to the results obtained, the trait total number of fruits presented a moderate seedling rootstock-mediated heritability of 0.36, indicating the possibility of harnessing natural variation from “criollo” plus trees *via* OP half-sib seedling rootstock families for cv. Hass orchards. On the contrary, the trait fruits’ weight presented a lower seedling rootstock-mediated heritability of 0.11, indicating that this trait may be influenced by environmental factors, non-additive genetic factors, such as dominance and epitasis, crop management, and the rootstocks-scion interaction, rather than additive genetic contributions from the OP half-sib rootstock families.

## Discussion

We quantified additive genetic parameters across landraces “plus trees” of the super-food fruit tree crop avocado (*P. americana* Mill.), and their OP half-sib seedling families. The SSR markers that were used for the molecular characterization of avocado “criollo” “plus trees” allowed obtaining *G*-BLUP heritability predictions of morphological and agronomic traits. After all, highly polymorphic SSR markers screened across variable genotypes (in terms of their degrees of relationships, from highly related to completely unrelated), enable BLUP predictions, as demonstrated by Reyes-Herrera et al. (2020) in avocado, and Cros et al. (2015, 2019) in oil palm and rubber tree, respectively. This is because pedigree-free marker-based heritability estimations *via G*-BLUP work either on the basis of shared relatedness (typically measured as relationships due to recent co-ancestry) or on the basis of genetic hitchhiking due to linkage disequilibrium (LD) among marker loci. In the absence of demonstrated LD (which would require denser single nucleotide polymorphism, SNP markers), shared relatedness as inferred by variable SSR markers across a diverse panel (Ellegren, 2004; Cortés et al., 2011) offer the basis to disclose the phenotypic clustering patterns across family types (which in turn are proportional to heritability scores). In particular, the relationship among samples is critically important, and therefore, was carefully reconstructed by our study *via* the relatedness histogram, the phylogenic relationships among samples, and the unsupervised Bayesian admixed clustering.

We estimated heritability values for 17 morpho-agronomic traits in avocado “criollo” “plus trees” selected as seed donors for rootstocks of the cv. Hass. These values were significant according to their 95% confidence intervals and heritabilities were found to be in a medium range according to Robinson et al. (1951) classification with the magnitude: *0.28* ≤ *h*^2^ ≥ *0.5*, indicating a moderate possibility of genetic gain through seedling rootstock selection and contribution of these traits to the cv. Hass. Regarding agronomic traits, the heritability of the fruit weight trait was quantified both in avocado “criollo” “plus trees” (0.36) and their OP half-sib seedling progenies used as rootstocks for the Hass scion (0.11), exhibiting a reduction of almost half in heritability. This indicates that nearly 50% of “plus tree” genetic gain for this trait may be inherited to cv. Hass through seed-mediated grafting.

### Leveraging Avocado “Criollo” “Plus Trees” as Seed Donors for Rootstock Production

A long-standing debate in the breeding of fruit tree crops is whether seedling rootstocks are capable to enhance productivity in the absence of more uniform clonal rootstocks, which in turn are thought to lack sufficient genetic variability for long-term adaptation. As an intermediate solution, replacing seedlings genotypes by a diverse panel of elite clones is expected to convey major improvements while controlling adaptive genetic erosion (Ingvarsson and Dahlberg, 2019). Nonetheless, avocado cv. Hass plantations in northwest South America have grown exponentially in the last decade without the availability of clonal rootstock genotypes. Instead, they have had to rely on cheaper seedling rootstocks from native “criollo” trees without any sort of selection or traceability. Therefore, our narrow sense heritability quantifications are encouraging guides to additive genetic selection of “criollo” “plus trees” as seed donors for rootstock production, conveying up to 35% (*h*^2^*_*T*_*) of potential genetic gain for a key yield trait as is the total number of fruits. This overall effect totalizes the additive genetic contribution from OP half-sib seedling rootstock families to the Hass clonal scion (i.e., *h*^2^*_*R*_* = 0.44, as computed by Reyes-Herrera et al., 2020), as well as the expected additive genetic variance putatively inherited from the “criollo” “plus trees” to their OP half-sib F1 seedling offspring (i.e., *h*^2^*_*F*1_* = *h*^2^*_*T*_*/*h*^2^*_*R*_* = 0.7). Ultimately, these estimates reinforce the feasibility of utilizing selected avocado “criollo” “plus trees” as seed donors for rootstocks.

Furthermore, the GEBVs, computed here by weighting and standardizing the overall trait superiority by the corresponding heritability scores, are already a robust selection index to target *in situ* superior “criollo” “plus trees” as donors of seedling rootstocks. Alternatively, buds from these selected “criollo” “plus trees” may be grafted to establish *ex situ* seed orchards. In any case, avocado cv. Hass nurseries may still face uncertainty while sorting the OP half-sib offspring from those “criollo” “plus trees” families in the absence of age-age correlations at the seedling stage. To assist early within-family selection of superior half-sib progenies, heritability estimates above 35% (e.g., for total number of fruits) also invite implementing last-generation high-throughput genomic selection (GS) schemes (Desta and Ortiz, 2014; Crossa et al., 2017). Genomic prediction platforms calibrate infinitesimal polygenic additive models (Cortés et al., 2020) capable to forecast breeding values for low-heritability complex traits (Klápště et al., 2020) at early life stages (Grattapaglia et al., 2018). Therefore, GS simultaneously makes indirect selection more precise, while allowing speeding up breeding cycles. Its potential in perennials with prolonged juvenile phases has already been industrially scaled up by the forest tree sector (Resende et al., 2012, 2020; Tan et al., 2017; Cappa et al., 2019; Thistlethwaite et al., 2019), with some prospective pilot studies carried out in temperate fruit trees (Kumar et al., 2012, 2019, 2020; Muranty et al., 2015; Iwata et al., 2016; Ferrão et al., 2021). Based on this, avocado cv. Hass nurseries might exploit the potential of GS to choose the superior progenies from the “criollo” “plus trees,” even before grafting. Such innovation will optimize timing and monetary resources at nurseries, while ultimately translating into reduced tree mortality and increased productivity/adaptability during the establishment and production phases.

Meanwhile, efforts must not be spared to breed a diverse panel of elite clonal rootstock genotypes resistant to root rot (*P. cinnamomi*), a major fungal threat for avocado plantation worldwide (Toapanta-Gallegos et al., 2017; Sánchez-González et al., 2019), while keeping high productivity and adaptation across heterogeneous mountain climates (Cortés and Wheeler, 2018). However, breeding elite clonal tree genotypes with conventional direct phenotypic selection doubles the breeding cycle length compared to gradual population recurrent selection due to longer progeny-testing and more clonal trials (Neale and Kremer, 2011). Therefore, GS may once more assist the identification of elite clonal rootstocks for the avocado cv. Hass industry, as already exemplified by the forestry sector by reducing the length of the clonal breeding cycle to 50% (Resende et al., 2012). Parallel efforts must be conveyed to better standardize the propagation of clonal rootstocks [e.g., *via* genotype-dependent micro-cloning (Ernst, 1999) and double grafting (Frolich and Platt, 1971) protocols], which is still a major bottleneck in regions with high availability of native avocado trees capable to source nurseries with cheaper seedlings.

### Paving the Path Toward Predictive Rootstock Breeding

The *h*^2^ and GEBVs estimate gathered as part of this study encourage the implementation of more systematic genetic-guided indirect selection platforms both as part of early (before grafting) seedling screening at nurseries of avocado cv. Hass, as well as within the avocado rootstock breeding cycles *per se*. Nonetheless, a question persists, which is whether the studied SSR molecular markers are enough to capture sufficient additive variance as to make predictive breeding reliable. This potentially remains open judging by the *r* scores (> 0.54) of our pedigree-free SSR-inferred estimation of genetic parameters (Frentiu et al., 2008; Wilson et al., 2010), which embraced sufficient variation across distinct kinship levels (Milner et al., 2000; Kruuk, 2004; Berenos et al., 2014). Therefore, avocado rootstock breeders would be able to leverage SSR characterizations across a diverse panel of avocado accessions (Alcaraz and Hormaza, 2007; Cañas-Gutiérrez et al., 2015; Ferrer-Pereira et al., 2017; Boza et al., 2018; Cañas-Gutiérrez et al., 2019b; Reyes-Herrera et al., 2020; Sánchez-González et al., 2020). SSR-inferred relationships also allow bridging the difficulty to generate complete pedigrees in tropical avocados. It is challenging to carry out control crossing in tropical “criollo” avocados because their pollination is open, and exhibit dichogamous protogyny. It would also be unviable to gather complete pedigrees from *in situ* “criollo” avocado trees because they exhibit disparity in the phenological phases, and are situated in remote locations where access is limited to perform controlled monitoring. This is why our team is currently bringing buds from the “criollo” trees for grafting on their own half-sib rootstocks. This strategy would allow cloning them at a single research station to better study their phenology and control their pollination. Meanwhile, molecular markers offer a realistic pathway to reconstruct relatedness among avocado “criollo” trees. The prospect to utilize highly polymorphic SSRs for predictive breeding across a diverse panel of genotypes spanning various levels of relatedness is also in line with previous proof-of-concept pilot studies carried out in oil palm (Cros et al., 2015) and rubber tree (Cros et al., 2019). This is because indirect selection works either on the basis of shared relatedness (typically measured as kinship relationships due to recent co-ancestry, Sedlacek et al., 2016) or on the basis of linkage disequilibrium (LD) between marker loci and the genetic variants that underlie phenotypic variation (Thistlethwaite et al., 2020). The high mutation rate of SSRs (Ellegren, 2004), and consequently, its unsurpassed polymorphism information content (Cortés et al., 2011; Blair et al., 2012), efficiently disclose the shared co-ancestry among genotypes (Lynch and Ritland, 1999) and its ultimate potential for predictive breeding.

Nonetheless, predictive breeding would be incapable to rely on LD (Blair et al., 2018) between markers and the genetic variants that underlie trait variation because SSRs are insufficient to reconstruct the polygenic architecture of complex (Hirschhorn and Daly, 2005) rootstock-mediated traits. After all, SSR markers, despite polymorphic, are rare across the genome, which makes them unlikely to be found in LD with any causal variant (Slatkin, 2008). To bridge this gap, future research will have to couple SNP screening of avocado genotypes (Kuhn et al., 2019; Rendón-Anaya et al., 2019; Rubinstein et al., 2019; Talavera et al., 2019), *via* genotyping-by-sequencing (GBS, Elshire et al., 2011; Cortés and Blair, 2018), whole-genome re-sequencing (WGR, Fuentes-Pardo and Ruzzante, 2017) and RNAseq (Jensen et al., 2012; Sun, 2012; Reeksting et al., 2016), with genome-wide association studies (GWAS, Khan and Korban, 2012). These innovative “omic” approaches should also target diverse avocado “criollo” “plus trees,” their OP half-sib progenies used as rootstocks for the Hass scion, and different tissues of the grafted tree, for instance through single-cell sequencing (Tang et al., 2019). Combined genetic mapping efforts will in turn inform on the upstream regulatory gene networks that contribute shaping the rootstock—scion compatibility (Loupit and Cookson, 2020) and interaction (Albacete et al., 2015; Warschefsky et al., 2016; Migicovsky and Myles, 2017), as well as on the corresponding downstream mechanistic physiological processes [e.g., transport of water and nutrients from the rootstocks, and large-scale movement of hormones, proteins, mRNAs, and sRNAs between the rootstock and the scion (Wang et al., 2017; Cookson et al., 2019)].

## Perspectives

The current research has proven the feasibility to transfer genetic superiority from native avocado “criollo” “plus trees” to commercial Hass plantations *via* OP half-sib seedling rootstock families. Specifically, our estimates suggest that this seedling rootstock breeding strategy may convey up to 35% of genetic gain for a relevant yield trait, as is the total number of fruits. Still, genetic-guided selection of “criollo” “plus trees” as donors of seedling rootstocks is also likely to impact other relevant traits both at the rootstock and scion levels. For instance, alterable rootstock phenotypes could include biotic resistance (Clavijo and Holguiín, 2020; Ramírez-Gil et al., 2020a,Guevara-Escudero et al., 2021) to root rot *P. cinnamomi* (Smith et al., 2011; Reeksting et al., 2016; Sánchez-González et al., 2019), drought and heat tolerance (López-Hernández and Cortés, 2019), mineral nutrient uptake (Bard and Wolstenholme, 1997; Calderón-Vázquez et al., 2013; Tamayo-Vélez and Osorio, 2018; Tamayo-Vélez et al., 2018), tolerance to salinity (Bernstein et al., 2001; Mickelbart and Arpaia, 2002; Raga et al., 2014), genetic compatibility (López-Guzmán et al., 2021), and harvest (Ramírez-Gil et al., 2020b) and postharvest physiochemical parameters (Astudillo and Rodríguez, 2018). This extended panel of potential rootstock effects is key for the avocado industry because cv. Hass lacks trait variation for resistance to diseases (e.g., it is susceptibility to *P. cinnamomi*, Sánchez-González et al., 2019). Therefore avocado cv. Hass is unable to provide a realistic source of rootstocks for commercial plantations, especially in soil types with a high natural incidence of *P. cinnamomi*. However, from a theoretical purely academic point of view, Hass grafted on Hass would be an appealing reference control treatment for future rootstock × scion factorial designs.

Meanwhile, indirect rootstock effects on scion’s traits may also involve alternate bearing and nutrition (Mickelbart et al., 2007), carbohydrate accumulation (Whiley and Wolstenholme, 1990), postharvest anthracnose development (Willingham et al., 2001), overall trunk high, and fruit production for the exportation market with acceptable fruit weight and lack of thrips’ damage (Reyes-Herrera et al., 2020). Therefore, future research efforts should aim quantifying, across this expanded repertoire of adaptive, morphological, harvest, and quality traits, combined *h*^2^ and GEBVs estimates that jointly account for the additive inheritance of phenotypic variance from the “plus trees” seedling donors to their OP progenies (i.e., family selection), as well as from these half-sib rootstock families to the avocado cv. Hass clonal scion (i.e., within-family offspring selection). These future studies should also envision collecting further data across years (beyond the 4 years production data for *in situ* “criollo” “plus trees”, and the 5 years production of the OP half-sib seedling rootstock progenies grafted with the cv. Hass clone). Altogether, these efforts will permit strengthening our modeling effort. Such baseline knowledge will further allow implementing predictive breeding platforms to guide before-grafting seedling selection at nurseries, while targeting diverse climates (Costa-Neto et al., 2020) as part of an “enviromic prediction” (Resende et al., 2020).

Diversifying selection of half-sib rootstock families and within-family offspring at seedling nurseries remains a promising and affordable avenue, especially if coupled with indirect genetic-guided predictors. Seedling rootstock breeding also enables broadening the genetic basis of young avocado plantations in regions with diverse sources of native avocado trees, some of them still cultivated in backyard gardens, traditional orchards, and as living fences (Galindo-Tovar et al., 2007). Yet, selection of diverse clonal rootstock genotypes may be a more appealing long-term strategy to contribute diminishing tree mortality by conveying genotypic superiority and phenotypic uniformity, while controlling genetic erosion (Ingvarsson and Dahlberg, 2019). Fulfilling the increased demand of diverse elite clonal rootstocks is becoming a pivotal requirement at avocado nurseries (Quintero et al., 2021), which could benefit by merging current rootstock breeding programs with predictive breeding platforms capable to boost selection accuracy of clonal rootstocks in shorter cycles (Resende et al., 2012).

## Conclusion

Our work has innovated the implementation of a pedigree-free marker-inferred heritability prediction model to assess the potential of avocado “criollo” “plus trees” as seed donors to produce OP half-sib rootstock families for the Hass clonal scion. The *h*^2^ scores and GEBVs gathered throughout this research indicate that the selection of seed donor “criollo” trees is able to confer, *via* seedling rootstocks, up to 35% of genetic gain for relevant yield traits, such as total number of fruits. This finding is in line with the overwhelming indirect rootstock effects on the avocado cv. Hass scion phenotype, which has been reported to impact a wide spectrum of harvest and quality traits beyond the total number of fruits (up to 44%), such as number of fruits with exportation quality, as well as those discarded because of low weight or thrips” damage. The feasibility to harness “criollo” seedling diversity within a rootstock-breeding program reinforces the utility of native avocado trees to source cryptic genetic variation and adaptive potential (Cortés and López-Hernández, 2021), ultimately counterbalancing the winnowing effect (Cortés et al., 2022) of clonal rootstocks and scions on genetic variation in fruit tree plantations.

## Data Availability Statement

The raw data supporting the conclusions of this article will be made available by the authors upon request, without undue reservation.

## Author Contributions

GC-G, AN-A, and AC conceived the original sampling and research questions. GC-G and SS-O performed phenotypic data collection and sampling of leaves for genotyping. GC-G carried out DNA extractions, SSR genotyping, and alleles size estimation. GC-G, FL-H, and AC filtered input datasets and carried out data analyses. FL-H and AC plotted diagrams. GC-G, FL-H, AN-A, and AC interpreted heritability scores. All authors contributed to the article and approved the submitted version.

## Conflict of Interest

The authors declare that the research was conducted in the absence of any commercial or financial relationships that could be construed as a potential conflict of interest.

## Publisher’s Note

All claims expressed in this article are solely those of the authors and do not necessarily represent those of their affiliated organizations, or those of the publisher, the editors and the reviewers. Any product that may be evaluated in this article, or claim that may be made by its manufacturer, is not guaranteed or endorsed by the publisher.

## Supplementary Material

Supplementary Figure 1 Distance tree computed by means of Neighbor Joining algorithm in R with 10,000 bootstrap replicates.

Supplementary Table 1 Avocado “criollo” “plus trees” considered as part of this study. ID number, municipality, sub-region within the province of Antioquia, and elevation are depicted in each case. A preliminary version of this table was originally compiled by Cañas-Gutiérrez et al. (2019b). Under the last column, the symbol + marks trees for which complete molecular and phenotyping characterizations were possible. Under the same column, the asterisk * marks “criollo” “elite trees” that donated seeds for the establishment of OP half-sib rootstock families grafted with the cv. Hass clone as part of the common garden (provenance trial) established at CI La Selva (Rionegro, Antioquia, 2,100 m asl).

Supplementary Table 2 Codification of qualitative traits (Table 1) measured in the avocado “criollo” selected “plus trees,” based on descriptors of the International Plant Genetic Resources Institute (IPGRI).

Supplementary Table 3 Primers and motif pattern for the 13 microsatellite markers (simple sequence repeats—SSRs) used in this study to avocado “criollo” “plus tree.” Forward and reverse primers, sequence motif, source, and summary statistics are shown. Markers were originally designed by Sharon et al. (1997) and Ashworth et al. (2004) and were prioritized according to their polymorphism information content (PIC), following Alcaraz and Hormaza (2007).
