# Supplementary figures and images for: Inheritance of Yield Components and Morphological Traits in Avocado cv. Hass From “Criollo” “Elite Trees” via Half-Sib Seedling Rootstocks
Source: Front Plant Sci. 2022 May 24;13:843099. doi: 10.3389/fpls.2022.843099 (PMC9171141; doi:10.3389/fpls.2022.843099)

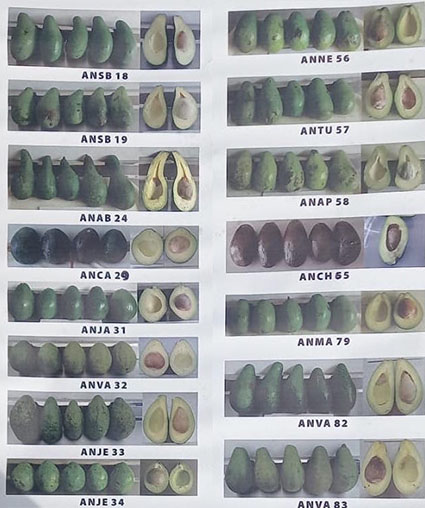

Supplement: Supplementary Figure 1 — Distance tree computed by means of Neighbor Joining algorithm in R with 10,000 bootstrap replicates. [file Image_1.jpeg]

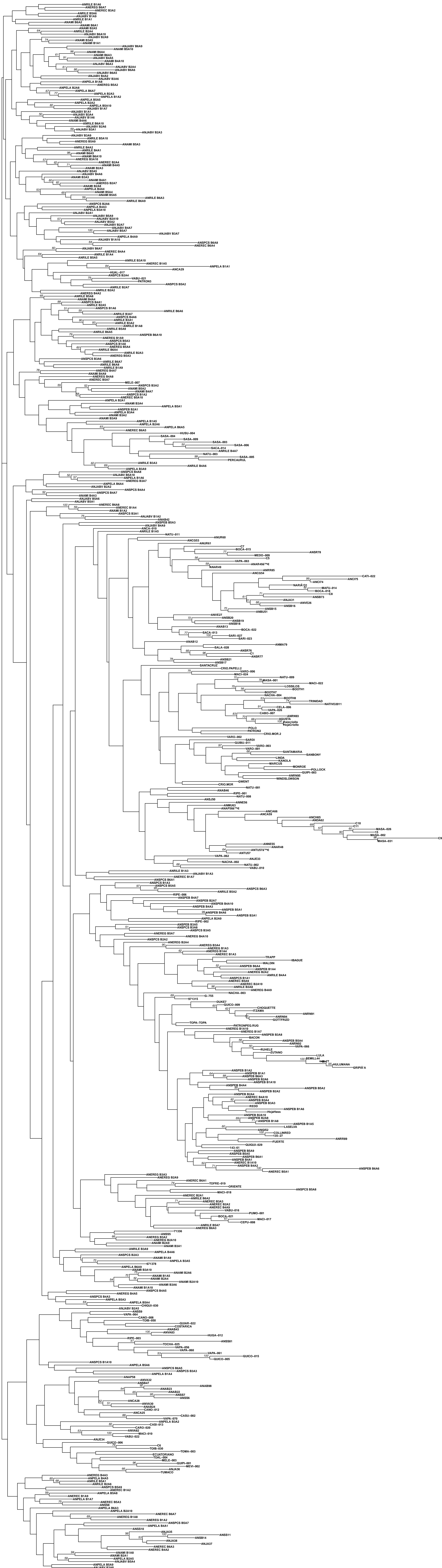

0.00 0.01 0.02 0.03 0.04

Genetic distance (proportion of loci that are different)

Supplement: Supplementary file 5 [file Data_Sheet_1.PDF]
